# Reply to Comment on "How Good is Your Model Fit? Weighted Goodness‐of‐Fit Metrics for Irregular Time Series"

**DOI:** 10.1111/gwat.13173

**Published:** 2022-01-21

**Authors:** R. A. Collenteur

**Affiliations:** ^1^ Institute of Earth Sciences, NAWI Graz Geocenter, University of Graz Graz 8010 Austria

## Introduction

I appreciate the comments by Dr. Zaadnoordijk (2022) and agree with the general notion that additional steps can be taken to improve the model shown as an example. This is also stated in the Supplementary material where the model is created and can be reproduced (Collenteur 2021b). The example data were used to illustrate the problem, independent of the model, and the weights were strictly applied to adapt the goodness‐of‐fit metrics to better deal with irregular time steps. The comments by Zaadnoordijk focus on obtaining a good model when dealing with irregular time series, whereas the original Commentary focused on the evaluation of a calibrated model. As such, I view them as a welcome extension of the discussion on how to deal with irregular time series during other phases of the modeling process. Below I respond to some of the comments by Zaadnoordijk.

## On the Applications of Noise Models

In the model presented in Collenteur (2021a) an AR(1) noise model was actually applied and irregular time steps were taken into account through its objective function (following von Asmuth and Bierkens 2005). However, as is clear from the example, we may still end up with a model that is biased toward the high (or even low) frequency period. The reason for this could be, for example, that the parameters ended up in a local optimum, or because a driving force is missing from the model. Regardless of the underlying reason, the example clearly shows that caution is required when interpreting fit metrics for irregular time series, as biases will occasionally still occur even when taking irregular time steps into account during calibration. In that case, we need to be able to identify such models and weighted fit metrics can help, particularly when doing large‐sample hydrology (e.g., analyzing thousands of wells).

Zaadnoordijk mentions that there is no need to compute goodness‐of‐fit metrics for a model, as long as the statistical assumptions made during model calibration are violated (e.g., residuals are ∼*N*(0,*σ* ^2^)). I generally agree with this statement, but note here that in practice the task of diagnostically checking the model assumptions is more difficult due to the same irregular time series. For example, for irregular time series it is still common practice to only check for autocorrelation at time lags that are (much) larger than the time step used for model calibration. This can lead to the misleading conclusion that no significant autocorrelation is present in the model residuals, while autocorrelation may still be present at smaller time lags. Solutions to compute autocorrelation for irregular time series are available (e.g., Rehfeld et al. 2011), but their usage may not be as widespread as they should be. Diagnostic checking remains a challenging task, and caution is therefore still required when interpreting goodness‐of‐fit metrics, even after checking the model assumptions.

## Improving the Model

After identification of a model with a poor fit, a next step could be to improve the model as suggested by Zaadnoordijk. While the model proposed by Zaadnoordijk clearly improves the visual fit, I think caution is required before accepting this alternative model for any prediction or decision support. The calibrated response functions for the additional precipitation and evaporation time series are extremely long (>8000 days), longer than the time series used for calibration (±7000 days). The resulting contributions from these stresses are uncertain, long‐term trends that are added to the model. However, the borehole is in the vicinity of a groundwater extraction well, and so a more obvious choice could be to improve model performance by better incorporating the pumping well into the time series model. A split‐sample test (e.g., by adding a validation period) could provide more information on the validity of alternative models.

## Concluding Remarks

Although it was not the purpose of the original contribution, the comments by Zaadnoordijk show the importance of taking irregular time steps into account in all modeling steps. More research into this topic is needed, for example by exploring the use of alternative weighting schemes such as the one proposed by Zaadnoordijk. Synthetic time series can be used to benchmark new weighting schemes and test alternative strategies to deal with irregular time steps in groundwater data.
